# Complete Genome Sequence of a Wild-Type Measles Virus Isolated during the Spring 2013 Epidemic in Germany

**DOI:** 10.1128/genomeA.00157-14

**Published:** 2014-04-17

**Authors:** Konstantin M. J. Sparrer, Stefan Krebs, Gundula Jäger, Sabine Santibanez, Annette Mankertz, Helmut Blum, Karl-Klaus Conzelmann

**Affiliations:** a Max von Pettenkofer-Institute, Ludwig-Maximilians-University, Munich, Germany; b Gene Center, Ludwig-Maximilians-University, Munich, Germany; c Laboratory for Functional Genome, Ludwig-Maximilians-University, Munich, Germany; d Robert-Koch Institute, National Reference Center Measles, Mumps, Rubella, Berlin, Germany

## Abstract

Measles virus induces an acute disease with rash and fever. Despite ongoing vaccination and elimination campaigns, the measles virus still sustains long-lasting transmission chains in Europe. Here we report the complete genome sequence of a wild-type measles virus isolated from a patient in Munich (MVi/Muenchen.DEU/19.13[D8]) during a German measles outbreak in 2013.

## GENOME ANNOUNCEMENT

Infections caused by measles virus (MV), the prototype of the *Morbillivirus* genus of the *Paramyxoviridae* family, remain a serious threat to human health worldwide. In 2011, approximately 160,000 deaths were caused by measles, although MV elimination is sought globally (1–3). Infection with MV is typically accompanied by transient immune suppression abetting secondary infections. MV itself can cause encephalitis and rare severe sequelae, including measles inclusion-body encephalitis (MIBE) and subacute sclerosing panencephalitis (SSPE), resulting inevitably in the death of the patient (4).

MV wild-type (wt) infection chains have been recently observed all over Europe (5, 6). In 2013, 704 measles cases were reported in Upper Bavaria and 306 in Munich. MV was isolated from a throat swab from a patient with typical clinical symptoms using Vero-hSLAM cells (7). The virus was cultivated for 48 h until syncytium formation was visible. Whole-cell RNA was extracted from infected Vero-hSLAM cells by using the Qiagen RNeasy minikit (Quiagen), and 100 ng was used to prepare a strand-specific RNA-Seq library kit (NuGEN Encore complete RNA-Seq library system; NuGEN, Inc.) following the manufacturer’s instruction. The resulting library was sequenced on an Illumina MiSeq instrument in paired-end mode with a read length of 250 nucleotides (nt).

Sequence reads were aligned at first to the sequence of the MV vaccine strain Schwarz (GenBank accession number AF266291.1) and for further refinement to that of a closely related MV isolate (MVi/Texas.USA/4.07, genotype D8; GenBank accession number JN635407.1). Only reads mapping to these MV strains were used for genome assembly using the *de novo* assembler Velvet (8). Coverage for an average nucleotide in the N gene was approximately 400 reads, and for the L gene, 50 reads. Nucleotides with a low coverage within the L gene and H-L gene border were sequenced using conventional reverse transcription and sequencing using specific primers (GATC, Constance, Germany). The nucleotide sequences of the 3′ leader and 5′ trailer regions were determined by 5′ rapid amplification of cDNA ends (5′-RACE) using specific primers binding in the leader and trailer sequences and terminal deoxynucleotidyl transferase (TdT) for addition of a poly(A) tail. PCR was carried out using a dT(18) primer with an EcoRI site, and the resulting DNA fragments were cloned into pCR3 vectors for sequencing of multiple clones. The presented full genome therefore represents an assembly of sequences obtained by the three techniques described above.

Genotyping according to the WHO protocol (9) using the 450 nucleotides of the 3′-end of the N gene classified the present wt strain as a D8 genotype, subtype Frankfurt-Main (MVs/Frankfurt Main.DEU/17.11-variant). The genome of MVi/Muenchen.DEU/19.13 follows the canonical 3′-N-P/V/C-M-F-H-L-5′ gene arrangement and comprises 15,984 nucleotides and therefore obeys the rule of six (10, 11). It shares 99% identity with the complete genome of the MVi/Texas.USA/4.07 wt isolate and 96.55% identity with that of the MV strain Schwarz. As is typically seen for wt versus vaccine strain comparisons, the C protein sequence contains a fully conserved nuclear localization sequence (NLS) (12), while the H protein lacks the typical N481Y or S546G mutation required for CD46 binding (13, 14). MVi/Muenchen.DEU/19.13 therefore represents a typical wt MV circulating in Germany.

### Nucleotide sequence accession number.

 The complete genome sequence of the MVi/Muenchen.DEU/19.13[D8] isolate is available at GenBank under the accession number KJ410048.

## References

[1] World Health Organization 2013 Measles reports. World Health Organization, Geneva, Switzerland http://www.who.int/mediacentre/news/notes/2013/measles_20130117/en/

[2] World Health Organization 2013 Measles fact sheet no. 286. World Health Organization, Geneva, Switzerland http://www.who.int/mediacentre/factsheets/fs286/en/

[3] World Health Organization 2013 Measles surveillance data. World Health Organization, Geneva, Switzerland http://www.who.int/immunization/monitoring_surveillance/burden/vpd/surveillance_type/active/measles_monthlydata/en/

[4] Griffin DE 2010 Measles virus-induced suppression of immune responses. Immunol. Rev. 236:176–189. 10.1111/j.1600-065X.2010.00925.x 20636817 PMC2908915

[5] Mankertz A Mihneva Z Gold H Baumgarte S Baillot A Helble R Roggendorf H Bosevska G Nedeljkovic J Makowka A Hutse V Holzmann H Aberle SW Cordey S Necula G Mentis A Korukluoglu G Carr M Brown KE Hubschen JM Muller CP Mulders MN Santibanez S 2011 Spread of measles virus D4-Hamburg, Europe, 2008–2011. Emerg. Infect. Dis. 17:1396–1401. 10.3201/eid1708.101994 21801615 PMC3381563

[6] Mankertz A Mulders MN Shulga S Kremer JR Brown KE Santibanez S Muller CP Tikhonova N Lipskaya G Jankovic D Khetsuriani N Martin R Gavrilin E 2011 Molecular genotyping and epidemiology of measles virus transmission in the World Health Organization European Region, 2007–2009. J. Infect. Dis. 204(Suppl 1):S335–S342. 10.1093/infdis/jir101 21666182

[7] Ono N Tatsuo H Hidaka Y Aoki T Minagawa H Yanagi Y 2001 Measles viruses on throat swabs from measles patients use signaling lymphocytic activation molecule (CDw150) but not CD46 as a cellular receptor. J. Virol. 75:4399–4401. 10.1128/JVI.75.9.4399-4401.2001 11287589 PMC114185

[8] Zerbino DR Birney E 2008 Velvet: algorithms for de novo short read assembly using de Bruijn graphs. Genome Res. 18:821–829. 10.1101/gr.074492.107 18349386 PMC2336801

[9] World Health Organization 2001 Nomenclature for describing the genetic characteristics of wild-type measles virus. Wkly. Epidemiol. Rec. 76:249–251 11561558

[10] Kolakofsky D Pelet T Garcin D Hausmann S Curran J Roux L 1998 Paramyxovirus RNA synthesis and the requirement for hexamer genome length: the rule of six revisited. J. Virol. 72:891–899 9444980 10.1128/jvi.72.2.891-899.1998 PMC124558

[11] Calain P Roux L 1993 The rule of six, a basic feature for efficient replication of Sendai virus defective interfering RNA. J. Virol. 67:4822–4830 8392616 10.1128/jvi.67.8.4822-4830.1993 PMC237869

[12] Sparrer KM Pfaller CK Conzelmann KK 2012 Measles virus C protein interferes with beta interferon transcription in the nucleus. J. Virol. 86:796–805. 10.1128/JVI.05899-11 22072748 PMC3255862

[13] Lecouturier V Fayolle J Caballero M Carabaña J Celma ML Fernandez-Muñoz R Wild TF Buckland R 1996 Identification of two amino acids in the hemagglutinin glycoprotein of measles virus (MV) that govern hemadsorption, HeLa cell fusion, and CD46 downregulation: phenotypic markers that differentiate vaccine and wild-type MV strains. J. Virol. 70:4200–4204 8676439 10.1128/jvi.70.7.4200-4204.1996 PMC190349

[14] Shibahara K Hotta H Katayama Y Homma M 1994 Increased binding activity of measles virus to monkey red blood cells after long-term passage in Vero cell cultures. J. Gen. Virol. 75 (Pt 12): 3511–3516.. 10.1099/0022-1317-75-12-3511 7996142

